# Pheromone production, male abundance, body size, and the evolution of elaborate antennae in moths

**DOI:** 10.1002/ece3.81

**Published:** 2012-01

**Authors:** Matthew RE Symonds, Tamara L Johnson, Mark A Elgar

**Affiliations:** 1 Department of Zoology, University of MelbourneVictoria 3010, Australia; 2 Centre for Integrative Ecology, School of Life and Environmental Sciences, Deakin UniversityBurwood, Victoria 3125, Australia.

**Keywords:** Antennal morphology, forewing length, Lepidoptera, phylogenetic generalized least squares, sex pheromone

## Abstract

The males of some species of moths possess elaborate feathery antennae. It is widely assumed that these striking morphological features have evolved through selection for males with greater sensitivity to the female sex pheromone, which is typically released in minute quantities. Accordingly, females of species in which males have elaborate (i.e., pectinate, bipectinate, or quadripectinate) antennae should produce the smallest quantities of pheromone. Alternatively, antennal morphology may be associated with the chemical properties of the pheromone components, with elaborate antennae being associated with pheromones that diffuse more quickly (i.e., have lower molecular weights). Finally, antennal morphology may reflect population structure, with low population abundance selecting for higher sensitivity and hence more elaborate antennae. We conducted a phylogenetic comparative analysis to test these explanations using pheromone chemical data and trapping data for 152 moth species. Elaborate antennae are associated with larger body size (longer forewing length), which suggests a biological cost that smaller moth species cannot bear. Body size is also positively correlated with pheromone titre and negatively correlated with population abundance (estimated by male abundance). Removing the effects of body size revealed no association between the shape of antennae and either pheromone titre, male abundance, or mean molecular weight of the pheromone components. However, among species with elaborate antennae, longer antennae were typically associated with lower male abundances and pheromone compounds with lower molecular weight, suggesting that male distribution and a more rapidly diffusing female sex pheromone may influence the size but not the general shape of male antennae.

## Introduction

**Figure 1:**
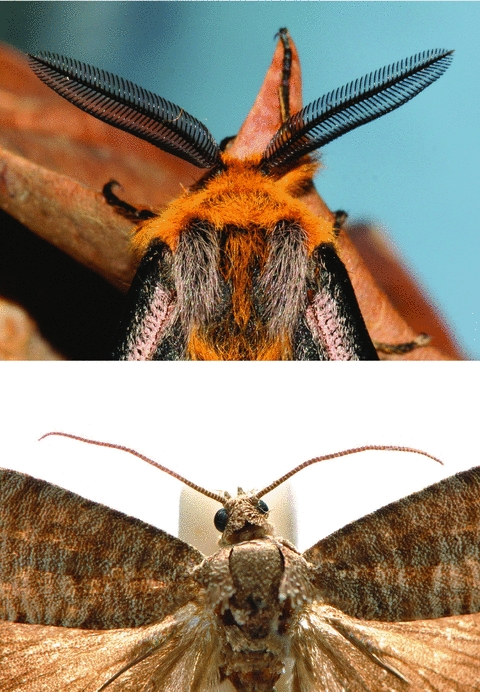
Antennal types: (a) elaborate bipectinate antennae of a male *Hemileuca eglanterina*, (b) simple filiform antennae of a male *Cydia pomonella*. Photos reproduced by kind permission: (a) Nicky Davis, http://www.wildutah.us, (b) Len Willan, CSIRO Entomology, http://www.csiro.au/resources/Australian-Moths.html

Moths are popularly characterized by two remarkable traits associated with chemical communication in a sexual context. First is the apparent ability of males to detect and respond to female sex pheromones over impressively long distances, including one anecdotal report of 11 km in an emperor moth, *Pavonia pavonia* (Regnier and Law 1968), even though females typically produce very small quantities of sex pheromone in the order of nanograms or even picograms (Greenfield 1981). Second, males of many species have beautiful and conspicuous feathery (i.e., “bipectinate” or “quadripectinate” comb-like—e.g., Fig. 1a) antennae (hereafter referred to as elaborate antennae), of which the most impressive examples are the Luna (*Actias luna*) and Hercules (*Coscinocera hercules*) moths.

These impressive biological receptor organs are usually found on males only, encouraging the view that elaborate antennae increase olfactory sensitivity to detect miniscule amounts of female pheromone in the atmosphere (Greenfield 1981; Birch and Haynes 1982; Cardé and Baker 1984; Rutowski 1984; Phelan 1992; Steinbrecht 1996; Svensson 1996). In insects, larger antennae are typically associated with a greater number of olfactory receptors (Chapman 1982) and a corresponding higher sensitivity to chemical signals (Birch and Haynes 1982; Chapman 1982; Spaethe et al. 2007). Such enhanced sensitivity to small amounts of sex pheromone detected over a long distance would be especially advantageous to males when there is strong competition for access to females, or if females are indirectly sexually selecting their mates by producing minute quantities in order to attract high-quality “sensitive” males (Lloyd 1979; Greenfield 1981). Additionally, elaborate antennae slow down and trap air-flow over the sensilla (e.g., Loudon and Koehl 2000), thereby potentially increasing the ability to detect scarce pheromone components in the atmosphere. Nevertheless, while many species of moths use long-distance sex pheromones (Greenfield 1981; Tamaki 1985; Cardé and Haynes 2004; El–Sayed 2011), relatively few moth species have elaborate antennae (Mankin and Mayer 1984), and most have simple filiform antennae (e.g., Fig. 1b) suggesting that the link between these characteristics is not necessarily straightforward. Accordingly, we test whether the long-held assumption that the evolution of elaborate antennae is linked to pheromone dynamics is supported by evidence from comparative data.

Elaborate antennae may provide an increased sensitivity to the pheromone by increasing the “active space” (Elkinton and Cardé 1984) of the signal: the area in which the concentration of the pheromone is above a threshold of detection and thus causes a behavioral response. Initial estimations of the size of this area were based on Bossert and Wilson's (1963) models of steady pheromone diffusion over time (see also Wyatt 2003, pp. 210–212). More recent research reveals that males detect pheromones by tracking wind-borne odor plumes that vary in concentration in space (Vickers 2006; Cardé and Willis 2008). Further, the turbulent nature of the environment (wind speed, temperature, local habitat structure, etc.) plays a critical role in shaping the response to pheromones (Elkinton and Cardé 1984; Byers 2008). While these environmental factors are vital to the immediate response of individual moths to pheromones, they cannot provide meaningful data from the perspective of the species, simply because there is no such thing as the average wind-speed for a species, for example. Nevertheless, the amount produced and composition of the sex pheromone, as well as population density, also determine the active space for detection, and are amenable to examination in a cross-species context. Thus, it is possible to derive means of testing whether elaborate antennae in male moths have evolved in response to the need for greater sensitivity to female sex pheromone.

We assessed the association between antennal morphology and aspects of their sexual chemical communication system using comparative data from 152 moth species. We started with two assumptions: (1) that the female sex pheromone signaling system will have evolved to attract at least one (but possibly more than one) suitable mate, driven by various processes of sexual selection; and (2) that the larger the active space of the pheromone plume then the greater the likelihood that there will be a male present that detects the pheromone. From these assumptions, we made the general prediction that elaborate antennae will have evolved to compensate against a reduction in the active space caused by some other property of the signaling system.

The first possibility is that the active space becomes smaller because females produce smaller quantities of pheromone. Thus, we predicted that females of species in which males have elaborate antennae should produce, on average, smaller amounts of pheromone than females of species in which males have more simple filiform antennae.

Second, we predict that active space becomes smaller if the pheromone components diffuse more quickly. Diffusion rates relate to both the generic type of compounds used (alkanes, alcohols, aldehydes, etc.), and to the molecular weight of the components of the pheromone blend (Butler and McDonough 1981; Ryan 1992). Moths typically use moderately volatile compounds, although there is variation in their diffusion rates, with molecular weights in the range of 200–300 (Wyatt 2003). We predict that elaborate male antennae will be more likely in species in which females use lower molecular weight compounds, which produce more rapidly dissipating signals.

The final prediction relates to population abundance. We predict that species with elaborate male antennae are more likely to be those characterized by low population abundance (hence a male is less likely to be within the active space of the female sex pheromone). In an analysis comparing antennal structure in two species of mantids (Holwell et al. 2007), the differences were explained using a similar argument that males require greater sensitivity to the volatile, long-distance mate attraction signals if the population exists at lower abundance. Here, we perform a wider comparative test of that prediction.

We also considered two other factors that may be important: body size and phylogeny. Body size is closely correlated with the length of antennal structures for many insects (e.g., Strauss 1990; Wcislo 1995; Kawano 2006), and so larger bodied species should similarly have larger (and perhaps more elaborate) antennae. This might particularly be so if there are aerodynamic costs associated with have larger, more unwieldy antennae at small body size (Ellington 1991). The relationship between pheromone titre and body size has only occasionally been investigated at the individual level, and then with varying results (e.g., Delisle and Vincent 2002; Ruther et al. 2009; Harari et al. 2011). Body size has well-documented negative relationships with population abundance (e.g., Currie 1993; White et al. 2007): smaller bodied species tend to have lower energetic demands and thus more individuals in a population can be sustained per unit area (Damuth 1981; Cotgreave 1993). Thus, in testing our principal hypotheses, we controlled for the possible confounding effect of body size.

The distribution of elaborate antennae among species is likely to be phylogenetically clumped. By mapping this trait onto a putative phylogeny, we estimated the number of evolutionary origins of this trait. More pertinently, closely related species may not necessarily provide independent data points in an analysis because they may share characteristics through common descent (Harvey and Pagel 1991). Therefore, we also control for phylogeny in our subsequent analyses.

## Methods

### Data collation

Information on antennal morphology of males was derived from published literature and field guides, as well as on-line lepidopterist resources. We categorized moth antennae unambiguously into two categories—simple and elaborate antennae. The former category covers moths whose antennae principally consist of a single shaft, which may be described as filiform, beadlike, ciliate, serrate, or dentate (e.g., Fig. 1b). Antennae were deemed elaborate if the antennal shaft branched off into side branches giving them a comb- or feather-like appearance, and may be described as pectinate, bipectinate, or quadripectinate (e.g., Fig. 1a). To provide a measure of body size, we collated information on the mean length of the forewing for males. This particular measure is the most commonly reported size parameter for Lepidoptera and regularly used in comparative analyses of the order (e.g., Gage 1994; Lindström et al. 1994; Summerville et al. 2006; Hambäck et al. 2007), and has been shown to act as a good proxy for body mass (*R*^2^≍ 80%; Miller 1977, 1997). Where the source gave a range for the length of the forewing (e.g., 14–20 mm), the midpoint of the range was taken as the value. We also collated data on antennal length, either directly from the literature, or by using length of forewing as a guide, and estimating length from pictures of male moths from the sources. Ideally, other measures of antennal area would also be employed (antennal width, overall surface area), but are not generally recorded in the literature and are difficult to estimate from pictures, therefore antennal length alone serves as our proxy for antennal size. However, in the analysis of antennal length, the moths were split into their two antennal morphology groups (simple or elaborate) and analyzed separately.

Information on pheromone titre was initially gathered from the Pherobase (El–Sayed 2011), cross-checking all information with the primary sources specified therein. Pheromone release rates have been identified for very few species, so pheromone titre, which is typically reported in most papers that analyze the pheromone composition, was used. We did, though, find a good association (*R*^2^ = 54%, *P* = 0.01) between pheromone release rate and pheromone titre in 10 species for which we had information on both measures (M. Symonds, unpubl. data). Pheromone titre is here described as the total quantity in nanograms of pheromone components that are functionally active (i.e., cause male attraction) present in the female gland. This value is averaged across the females chemically sampled, generating a measure of nanogram per female. In addition to the total quantity of components, we also carried out the analysis using amount of the major and most minor components as our measure of pheromone titre, but it had no effect on our conclusions (data not shown).

Data on the molecular weights of the functionally active compounds in the pheromone blend was also taken from the Pherobase. We calculated a mean molecular weight of these components in cases where more than one compound was identified as active. Analyses using maximum and minimum molecular weights produced qualitatively the same results and are not shown.

Estimating population densities or abundance is challenging, especially from pheromone trap data because many factors (climatic conditions, seasonality, number of traps, trap spacing, concentration and quality of pheromone, host plants, use of insecticides, etc. see McNeil 1991 for a review) can affect male responses. Light-trapping data are similarly problematic because they provide poor estimates at low populations densities and light-traps do not necessarily attract moths at the time (seasonally or daily) at which females are calling (Delisle et al. 1998). In addition, fewer data on population density from light trapping exist for the species in our analysis. Accordingly, we use the pheromone trapping information taken primarily from the same sources that described pheromone composition and titre—this having the added advantage of abundance estimates being directly taken from the same populations as our pheromone data. We noted the highest recorded trapping intake observed for that population of moths (standardized across papers as the number of males caught per trap per night). We considered this a better measure of male abundance than mean values from these papers since not all of the trapping experiments reported in a study use optimal pheromone compositions. Also male abundance can vary greatly even during the course of a season, making mean abundance a less reliable measure. Maximum trapping intake should therefore more closely reflect the actual number of males within the “active space” of the pheromone at the time when females are most likely to be calling. In order to account for differences in trapping protocol across papers, we also calculated a second measure of abundance that took into account the number and distance apart of the traps: specifically we calculated the residuals from the model predicting the log number of individuals trapped per trap night using the predictors of log number of traps and log minimum distance between traps. In practice, the absolute and relative measures of abundance gave qualitatively the same results, so we report results using the absolute measure only. The dataset is presented in Table 1.

**Table 1:** The dataset used in the analyses. ANT, antenna type; AL, antenna length (mm); FL, forewing length (mm); Q, pheromone titre (ng); MW, mean molecular weight of pheromone components; *k*_males_, abundance of males (maximum number of males caught per trap per night). Pheromone data were taken from El–Sayed (2011) and references therein, as were male abundance (trapping) data, except where indicated in the references (*). References for additional natural history data on antennal morphology and forewing length are also given

Family	Species	ANT	AL	FL	Q	MW	*k*_males_	Refs.
Acrolepiidae	*Acrolepiosis assectella*	Simple	3.54	5.90	1.00	238.41	10.70	6, 93*
Arctiidae	*Holomelina lamae*	Simple	2.61	9.00	7498.0	259.18		58
Arctiidae	*Panaxia quadripunctaria*	Simple	10.34	23.50	10100	285.54		9
Arctiidae	*Pyrrharctia isabella*	Simple	7.15	27.50		254.50		80
Arctiidae	*Utetheisa ornatrix*	Simple	9.02	22.00	32.00	290.53	4.70	80
Argyresthiidae	*Argyresthia conjugella*	Simple	2.34	6.00	1.10	282.47	10.10	9
Bombycidae	*Bombyx mori*	Elaborate	6.80	20.00	210.90	237.41		21
Carposinidae	*Carposina sasakii*	Simple	4.93	8.50	1.67	287.51	3.75	26
Carposinidae	*Coscinoptycha improbana*	Simple	3.75	7.50	69.80	301.54	0.45	36
Cossidae	*Cossus cossus*	Elaborate	17.21	46.50	151.50	240.39	2.43	8
Cossidae	*Holcocerus insularis*	Simple	9.69	19.00	14.40	226.38		18
Crambidae	*Deanolis sublimbalis*	Simple	6.80	10.00	0.48	278.50	4.24	40
Crambidae	*Eoreuma loftini*	Simple	6.10	10.00	56.30	286.49	6.10	5
Crambidae	*Glyphodes perspectalis*	Simple	13.80	20.00	86.00	238.41	0.80	18, 52
Crambidae	*Glyphodes pyloalis*	Simple	5.40	10.00	2.00	278.43	24.64	5
Cramgbidae	*Ostrinia furnacalis*	Simple	8.53	13.75	12.40	254.41	6.07	23, 50
Crambidae	*Ostrinia latipennis*	Simple	7.53	14.20	3.80	212.37	1.70	18, 72
Crambidae	*Ostrinia palustralis*	Simple	7.88	17.50	37.50	254.41	0.96	35
Crambidae	*Ostrinia zaguliaevi*	Simple	8.24	13.50	22.80	254.41		18
Eriocranidae	*Eriocrania cicatricella*	Simple	2.21	4.90	159.00	115.20	29.60	47
Gelechiidae	*Anarsia lineatella*	Simple	4.25	6.25	283.40	177.29		8
Gelechiidae	*Keiferia lycopersicella*	Simple	2.57	5.25	10.10	240.39	29.40	5, 49
Gelechiidae	*Pectinophora gossypiella*	Simple	6.13	8.75	10.00	280.45		76
Gelechiidae	*Phthorimaea operculella*	Simple	4.81	7.75	12.50	237.36	61.00	24, 49
Gelechiidae	*Scrobipalpa ocellatella*	Simple	4.02	5.75	70.00	226.36		8
Gelechiidae	*Tuta absoluta*	Simple	2.88	4.50	5.44	251.39	810.00	91, 102*
Geometridae	*Abraxas grossulariata*	Simple	7.49	20.25	0.55	242.41	2.82	1
Geometridae	*Alsophila pometaria*	Simple	6.45	15.00	46.20	261.13	10.40	10, 11
Geometridae	*Ascotis selenaria*	Elaborate	7.96	21.50	42.00	278.48		18, 19
Geometridae	*Ascotis selenaria cretacea*	Elaborate		21.50	35.00	270.48	16.00	18, 19
Geometridae	*Erannis defoliaria*	Elaborate	7.30	18.25	2.00	270.48	0.15	42, 46
Geometridae	*Eupithecia assimilata*	Simple	8.93	19.00	23.00	306.53	0.17	1
Geometridae	*Idaea aversata*	Simple	7.41	14.25		238.37	0.07	1
Geometridae	*Lambdina athasaria*	Elaborate	4.05	13.50	0.06	261.52	10.00	5
Geometridae	*Milionia basalis pryeri*	Simple	15.82	28.25	7.00	278.48	29.00	67
Geometridae	*Mnesampela privata*	Simple	11.80	20.00	110.00	262.48	1.32	24, 68
Geometridae	*Operophtera bruceata*	Simple	6.09	14.50	1.00	260.46	28.78	69
Geometridae	*Operophtera brumata*	Simple	4.73	11.25	1.00	260.46	11.84	63
Geometridae	*Peribatodes rhomboidaria*	Elaborate	9.20	20.00	1.25	270.48	1.63	42
Geometridae	*Sabulodes caberata*	Simple	12.00	25.00	37.50	264.49	2.06	5, 21
Geometridae	*Tephrina arenacearia*	Elaborate	6.38	12.50	4.00	250.40		87
Gracilariidae	*Caloptilia porphyretica*	Simple		7.50	6.00	238.41	37.10	8, 25
Gracilariidae	*Conopomorpha cramerella*	Simple	6.24	6.00	0.10	254.42	12.93	32, 33
Gracillariidae	*Phyllonorycter mespilella*	Simple	2.80	3.50	1.00	224.34	190.00	77, 78
Gracillariidae	*Phyllonorycter ulmifoliella*	Simple	2.80	4.00	1.00	254.41	39.30	9, 79
Incurvariidae	*Lampronia capitella*	Simple	4.03	7.75	15.00	223.70	12.73	9, 62
Lasiocampidae	*Malacosoma neustrium*	Elaborate	6.67	14.50	143.10	181.30	1.21	8, 18
Limacodiidae	*Parasa lepida*	Elaborate	10.40	20.00	20.00	154.25	2.00	75
Lymantriidae	*Artaxa subflava*	Elaborate	4.21	14.50	10.00	326.56	0.63	18
Lymantriidae	*Euproctis pseudoconspersa*	Elaborate	2.64	12.00	10.00	326.56	19.60	50
Lymantriidae	*Euproctis pulverea*	Elaborate	4.11	13.25	270.00	376.61	0.21	51
Lymantriidae	*Orgyia leucostigma*	Elaborate	5.85	15.00	5.00	306.53	1.03	13, 70
Lymantriidae	*Orgyia postica*	Elaborate	4.46	13.50	30.70	306.53	2.40	71
Lymantriidae	*Perina nuda*	Elaborate	4.55	17.50	263.00	317.20	7.60	18, 71
Lymantriidae	*Teia anartoides*	Elaborate	4.00	10.00	57.29	300.55	0.90	23, 24
Lyonetiidae	*Lyonetia clerkella*	Simple	3.38	4.50	100.00	266.51	116.00	8, 41
Tineidae	*Tineola bisselliella*	Simple	5.06	6.25	0.53	265.46	0.82	9, 90, 101*
Torticidae	*Cnephasia longana*	Simple	5.30	10.00	0.10	226.36	0.69	8, 14
Noctuidae	*Agrotis ipsilon*	Elaborate	14.63	22.50	0.21	254.41	2.70	8, 13
Noctuidae	*Autographa gamma*	Simple	15.00	20.00	2.20	205.34	0.55	8, 20, 94*
Noctuidae	*Brithys crini*	Simple	9.60	20.00	600.00	238.41		23, 24
Noctuidae	*Copitarsia decolora*	Simple	8.69	16.10	7.55	233.39	3.97	34
Noctuidae	*Cornutiplusia circumflexa*	Simple	9.03	21.00	2.82	205.34	3.27	9, 35
Noctuidae	*Earias insulana*	Simple	5.52	12.00	1.00	236.40	3.14	43, 44
Noctuidae	*Earias vittella*	Simple	5.20	10.00	40.00	247.09	176.50	9
Noctuidae	*Epiglaea apiata*	Simple	11.70	19.50	2.00	264.44	6.13	5
Noctuidae	*Euxoa messoria*	Simple	12.38	18.75	10.40	268.46	26.00	21
Noctuidae	*Euxoa ochrogaster*	Simple	9.68	19.75	12.49	226.36	4.78	21
Noctuidae	*Graphania mutans*	Elaborate	23.60	40.00	4.63	233.39	1.97	53
Noctuidae	*Helicoverpa armigera*	Simple	10.40	20.00	46.72	238.41	8.40	8, 23
Noctuidae	*Helicoverpa assulta—Korea*	Simple	7.38	12.50	421.00	260.44	18.75	23, 24
Noctuidae	*Helicoverpa assulta—Thailand*	Simple	7.38	12.50	161.28	238.41	2.09	23, 24
Noctuidae	*Helicoverpa peltigera*	Simple	11.02	19.00	58.30	229.73	10.20	56
Noctuidae	*Helicoverpa punctigera*	Simple	16.40	20.00	10.00	253.77	20.50	24, 40
Noctuidae	*Helicoverpa virescens*	Simple	9.14	15.75	169.31	229.73	1.56	10, 57, 97*
Noctuidae	*Helicoverpa zea*	Simple	10.40	20.00	23.53	238.41	12.00	8
Noctuidae	*Lacinipolia renigera*	Simple	7.27	12.75	8.10	253.41	5.65	37, 61
Noctuidae	*Mocis latipes*	Simple	7.80	20.00	20.06	291.54		5
Noctuidae	*Nephelodes minians*	Elaborate	10.63	21.25	134.30	260.44	1.30	5, 21
Noctuidae	*Oraesia excavata*	Elaborate	7.27	24.25	130.00	307.54		18
Noctuidae	*Panolis flammea*	Simple	9.18	17.00	53.00	263.76		8, 73
Noctuidae	*Peridroma saucia*	Simple	14.10	23.50	65.00	268.44	3.20	8
Noctuidae	*Sesamia grisescens*	Simple	5.95	17.50	85.93	261.45	2.00	40
Noctuidae	*Sesamia nonagrioides*	Elaborate	6.97	17.00	33.79	253.77	44.10	8, 81
Noctuidae	*Spodoptera eridania*	Simple	21.60	36.00	3.50	258.82	14.30	82
Noctuidae	*Spodoptera littoralis*	Simple	11.34	18.00	13.37	252.40	345.00	8, 99*
Noctuidae	*Thysanoplusia orichalcea*	Simple	13.00	20.00	22.20	225.35	1.23	9, 89
Noctuidae	*Trichoplusia ni*	Simple	11.03	17.50	69.19	236.05	2.66	9
Noctuidae	*Tyta luctuosa*	Simple	6.76	13.00	139.00	224.39		84
Nolidae	*Uraba lugens*	Elaborate	5.91	13.75	19.50	259.43	0.41	23, 92
Oecophoridae	*Cheimophila salicella*	Simple	5.70	9.50	9.50	232.39	29.70	27, 95*
Plutellidae	*Homadaula anisocentra*	Simple	3.43	7.00	10.00	254.41		59
Plutellidae	*Plutella xylostella*	Simple	2.87	7.00	0.37	253.77	56.00	8, 23
Psychidae	*Thridopteryx ephemeraeformis*	Elaborate	4.37	13.25	375.00	242.40	27.30	5, 15
Pyralidae	*Acrobasis nuxvorella*	Simple	7.75	12.50	0.002	258.43	1.23	2, 3
Pyralidae	*Acrobasis vaccinii*	Simple	5.27	8.50	0.52	267.43	11.25	4, 5
Pyralidae	*Etiella behrii*	Simple	4.73	10.50	0.23	240.89	1.10	48
Pyralidae	*Etiella zinckenella (Europe)*	Simple	6.75	11.25	14.90	254.92	0.91	23, 49
Pyralidae	*Etiella zinckenella (Japan)*	Simple	6.75	11.25	6.80	245.06	1.90	23, 49
Pyralidae	*Homoeosoma nebulellum*	Simple	6.70	11.75	9.52	237.74	3.10	9
Pyralidae	*Plodia interpunctella*	Simple	6.12	9.00	26.30	231.38	3.93	8, 23, 98*
Saturniidae	*Coloradia velda*	Elaborate	14.62	39.50	2.79	266.44	1.80	5, 30, 31
Saturniidae	*Hemileuca eglanterina*	Elaborate	10.64	38.00	84.99	251.75	2.11	5, 30, 31
Saturniidae	*Hemileuca maia*	Elaborate	9.38	31.25	22.74	251.75	1.46	5, 30, 31
Sesiidae	*Macroscelesia japona*	Elaborate	5.78	10.50	4.50	265.46	1.81	18
Sesiidae	*Macroscelesia longipes*	Elaborate	5.57	10.50	17.20	265.46	3.35	18
Sesiidae	*Paradoxecia pieli*	Elaborate	5.27	13.50	250.00	308.50	5.50	74
Sesiidae	*Synanthedon exitiosa*	Simple	9.75	16.25	100.00	308.50		85
Sesiidae	*Synanthedon pictipes*	Simple	6.66	10.25	4.00	308.50	111.25	28, 86
Sphingidae	*Agrius convolvuli*	Simple	22.50	50.00	7.00	236.40		12
Sphingidae	*Manduca sexta*	Simple	25.80	53.75	15.20	235.39	16.00	64, 65
Stathmopodidae	*Stathmopoda masinissa*	Simple	5.11	7.00	0.05	280.45	5.71	83, 84
Thaumetopoeidae	*Thaumetopoea pityocampa*	Elaborate	7.82	17.00	1.00	278.43	2.39	8, 100*
Tortricidae	*Adoxophyes orana*	Simple	4.08	8.50	186.40	233.39	4.95	7, 8
Tortricidae	*Agapeta zoegana*	Simple	5.40	10.00	8.00	254.41		9
Tortricidae	*Archips breviplicanus*	Simple	4.50	10.00	28.90	254.41	15.95	14
Tortricidae	*Archips semiferana*	Simple	5.46	9.75	33.33	254.41	62.10	5, 15
Tortricidae	*Argyrotaenia pomililiana*	Simple	3.65	7.30	3.16	232.39	1.77	16
Tortricidae	*Argyrotaenia velutinana*	Simple	2.80	6.50	115.00	245.73	2.45	17
Tortricidae	*Bonagota salubricola*	Simple	2.85	7.50	2.71	246.39	0.50	22
Tortricidae	*Choristoneura conflictana*	Simple	5.85	15.00	18.00	210.36	11.00	5, 28
Tortricidae	*Choristoneura retiniana*	Simple	5.58	11.63	23.00	233.39	43.00	5
Tortricidae	*Cnephasia jactatana*	Simple	4.96	8.70	1.80	254.41	1.88	29
Tortricidae	*Croesia curvalana*	Simple	4.05	7.50	0.16	232.39	91.80	37, 38
Tortricidae	*Cryptophlebia amamiana*	Simple	3.30	7.50	6.00	226.36		39
Tortricidae	*Cryptophlebia horii*	Simple	4.29	8.75	4.00	184.32	9.05	39
Tortricidae	*Ctenopseustis herana*	Simple	4.44	12.00	3.30	254.41	0.75	40
Tortricidae	*Ctenopseustis obliquana*	Simple	6.00	12.00	1.25	254.41		40
Tortricidae	*Cydia caryana*	Simple	2.64	5.50	0.03	224.34	13.81	5, 21
Tortricidae	*Cydia pomonella*	Simple	6.82	11.00	3.39	197.94	18.00	8, 41
Tortricidae	*Cydia pyrivora*	Simple	4.31	10.50	0.50	224.34	0.26	41, 42
Tortricidae	*Cydia splendana*	Simple	5.13	9.00	0.09	224.34		8, 14
Tortricidae	*Endopiza viteana*	Simple	2.05	5.00	1.44	240.39	0.68	5, 45
Tortricidae	*Epinotia tedella*	Simple	1.56	6.50	0.40	224.34	4.39	8, 14
Tortricidae	*Eupoecilia ambiguella*	Simple	2.31	7.00	2103.0	255.75	4.67	8, 14, 96*
Tortricidae	*Grapholita dimorpha*	Simple	2.53	5.38	3.40	226.36	0.62	54, 55
Tortricidae	*Grapholita funebrana*	Simple	2.69	6.25	0.51	226.36	1.23	9, 41
Tortricidae	*Homona magnanima*	Simple	6.44	11.50	160.00	235.71	62.40	14, 18
Tortricidae	*Homona spargotis*	Simple	2.35	8.38	8.90	215.15	1.10	60
Tortricidae	*Lobesia botrana*	Simple	2.82	6.00	0.36	219.20	2.00	7, 8, 63
Tortricidae	*Melissopus latiferreanus*	Simple	4.50	9.00	2.00	224.34	2.18	15, 66
Tortricidae	*Platynota idaeusalis*	Simple	4.16	9.25	60.00	233.39		15
Tortricidae	*Rhopobota naevana*	Simple	3.85	7.00	0.13	212.37	14.04	7
Tortricidae	*Sparganothis pilleriana*	Simple	3.71	9.50	0.22	245.06	3.11	5, 8, 14
Tortricidae	*Thaumatotibia batrachopa*	Simple		8.25	9.95	226.36	2.03	40, 88
Tortricidae	*Thaumatotibia leucotreta*	Simple	4.29	8.25	296.00	226.36	0.93	40
Tortricidae	*Tortrix viridana*	Simple	5.61	11.00	4.00	254.41		8
Yponomeutidae	*Yponomeuta cagnagellus*	Simple	6.89	11.30	5.32	255.08	4.76	7, 8, 103*
Yponomeutidae	*Yponomeuta evonymellus*	Simple	6.39	10.30	3.92	240.40	1.10	8
Yponomeutidae	*Yponomeuta padellus*	Simple	7.04	11.00	13.56	263.76	2.02	7, 8, 103*
Yponomeutidae	*Yponomeuta plumbellus*	Simple	6.39	9.00	1.24	254.41	2.62	7, 8, 103*
Yponomeutidae	*Yponomeuta rorellus*	Simple	6.59	10.80	5.00	256.43	0.13	8, 103*

^1^Skou (1986), ^2^United States Department of Agriculture (2011), ^3^Mulder and Grantham (2003), ^4^IPM North Carolina (1997a), ^5^North American Moth Photographers Group (2007), ^6^Landry (2007), ^7^Gustaffson (2003), ^8^Carter (1984), ^9^[Bibr b178][Bibr b178], ^10^Canadian Biodiversity Information Facility (2011), ^11^Hoover and Haydt (2001), ^12^Pittaway and Kitching (2000–2011), ^13^Tumlison and Benjamin (2011), ^14^Meijerman and Ulenberg (2000), ^15^BugGuide.Net (2011), ^16^Trematerra and Brown (2004), ^17^The Virginia Fruit Page (2011), ^18^Jpmoth.org (2011), ^19^Forbes (1925), ^20^Venette et al. (2003), ^21^Arnett (2000), ^22^Brown and Razowski (2003), ^23^Australian Moths Online (1994–2011), ^24^Herbison–Evans and Crossley (2011), ^25^Zhang and Polavarapu (2004), ^26^Anonymous (2011), ^27^Medvedev (1990), ^28^E. H. Strickland Entomological Museum (2001–2011), ^29^Jiménez–Pérez and Wang (2004), ^30^Tuskes et al. (1996), ^31^Butterflies and Moths of North America (2011), ^32^Menzel and Waite (2005), ^33^Rauf (2008), ^34^Simmons and Pogue (2004), ^35^Jonko (2011), ^36^Hoare et al. (2011), ^37^Line (2007), ^38^Crozier (1996), ^39^Komai and Nasu (2003), ^40^Pest and Diseases Image Library (2011), ^41^Afonin et al. (2008), ^42^Alford (2007), ^43^BioLib (1999–2011),^44^Melifronides et al. (1978), ^45^Williams et al. (2011), ^46^Ramel (2011), ^47^Kurz and Kurz (2000–2011), ^48^Brier (2010), ^49^King and Saunders (1984), ^50^Korean Natural History Research Information System (2011), ^51^Insects of Japan (2011), ^52^Korycinska and Eyre (2011), ^53^Dugdale (1971), ^54^Bae and Park (1997), ^55^Komai (1979), ^56^National Museums Northern Ireland (2009–2011), ^57^Featured Creatures (1996–2011), ^58^Cardé (1965), ^59^Heppner and Dekle (1975), ^60^Whittle et al. (1987), ^61^Hants Moths Group (2011), ^62^Stichting TINEA (2011), ^63^Fraval (2011), ^64^Oehlke (2011), ^65^Schneider et al. (1997), ^66^Scott (2001–2011), ^67^National Taiwan University Insect Museum Digital Archives Project (2011), ^68^Elliott and Bashford (1978), ^69^Miller and Hammond (2000), ^70^Natural Resources Canada (2009), ^71^Mohn (1993–2005), ^72^Ohno et al. (2003), ^73^Savela (2011), ^74^Gorbunov and Arita (1997), ^75^Waller et al. (2007), ^76^Hill (2008), ^77^Papillons de Poitou–Charentes (2011), ^78^Norfolk Moths (2011), ^79^Association Lepiforum (2011), ^80^Conner (2008), ^81^Israeli Ministry of Agriculture and Rural Development (2011), ^82^IPM North Carolina (1997b), ^83^Naka et al. (1998), ^84^Watson and Dallwitz (2003–2011), ^85^Duckworth and Eichlin (1977), ^86^Hogmire (1995), ^87^Berlov and Berlov (1999–2011), ^88^United States Department of Agriculture (2010), ^89^Holloway (1985), ^90^Western Australian Department of Agriculture (2011), ^91^Russell IPM (2011), ^92^Phillips (1992), ^93^Renou et al. (1981), ^94^Tóth et al. (1983), ^95^Salas–Reyes (1985), ^96^Rauscher et al. (1984), ^97^Dickens et al. (1993), ^98^Doud and Phillips (2000), ^99^Kehat et al. (1976), ^100^Quero et al. (2003), ^101^Cox et al. (1996), ^102^Ferrara (2001), ^103^Löfstedt and Herrebout (1988).

### Phylogenetic information

**Figure 2:**
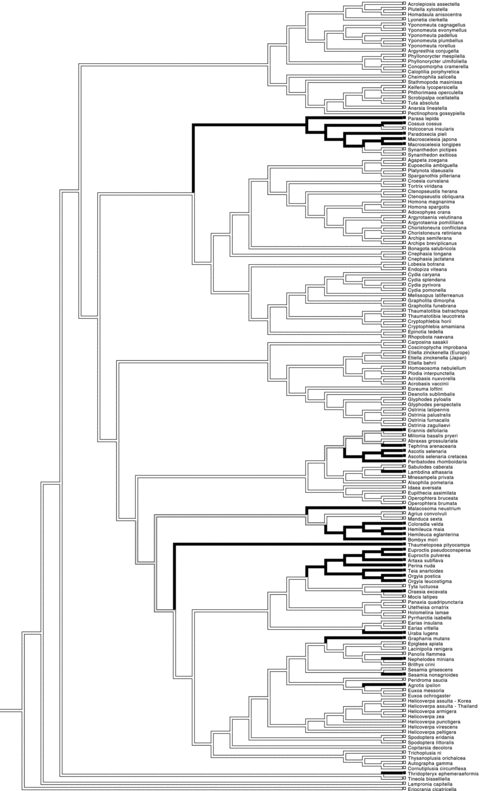
Phylogeny used in the analysis. Lineages leading to species with elaborate antennae are marked on in black. Putative reconstruction of evolutionary transitions is based on maximum parsimony analysis in Mesquite (Maddison and Maddison 2010).

We constructed a composite phylogeny (see Fig. 2) combining phylogenetic information from a number of sources as follows: The species were initially split based on taxonomy down to the generic level. Relationships between superfamilies were derived from Kristensen et al. (2007) with further resolution of relationships from Minet (1991) (Gelechoidea, Yponomeutoidea, Cossoidea, Sessoidea, and Zygaenoidea) and Regier et al. (2008) (Geometroidea, Noctuoidea, Lasiocampoidea, and Bombycoidea). Relationships within the Geometroidea were taken from Yamamoto and Sota (2007), with further resolution of the position of *Peribatodes* and *Ascotis* from Hunter (1995), and *Tephrina* from Young (2008). Bombycoidea internal relationships were derived from Regier et al. (2008), while Gelechioidea phylogeny was derived from Kaila (2004) with additional resolution for the Gelechiidae from Lee et al. (2009). Solis (2007) provided the phylogeny for the Pyraloidea, with additional resolution within the Pyralidae from Simonsen (2008) and within *Ostrinia* from Ishikawa et al. (1999). The phylogeny of the Noctuoidea was taken from Mitchell et al. (2006) with further resolution within the Lymantriidae and the Hadeninae from Lafontaine and Fibiger (2006) and within the Heliothinae from Cho et al. (2008). Relationships between genera in the Yponomeutoidea were derived from the systematic arrangement proposed by Dugdale et al. (1999), and further resolution within the genus *Yponomeuta* was taken from Löfstedt et al. (1991). Finally, family and subfamily relationships within the Tortricoidea were taken from Roelofs and Brown (1982), with resolution within the Archipini derived from Pashley (1983), Lee et al. (2005), Hulcr et al. (2007), and Safonkin and Triseleva (2008), and relationships within the Grapholitini from Pashley (1983) and Komai (1999).

The composite nature of the phylogeny means that branch length information was not available, and so all branch lengths were set to the same length (= 1).

### Data analysis

To meet assumptions of normality, all continuous variables except mean molecular weight were log-transformed before inclusion in the analysis. Relationships between aspects of antennal morphology, body size (forewing length), and the main variables of interest (pheromone titre, mean molecular weight, and population abundance) were determined controlling for phylogenetic relatedness. This was achieved using phylogenetic generalized least squares (PGLS) (Martins 1996; Martins and Hansen 1997), implemented through the package COMPARE (Martins 2004). PGLS is a statistical method that allows one to investigate the correlation between continuous variables and a limited number of categorical variables (as predictor variables) across species. By comparing the observed covariance in traits with that expected under a specified model of evolution (in this case a Brownian motion model) it can calculate this correlation controlling for the phylogenetic signal in the traits being analyzed (expressed in terms of the variable α, where low values tending to 0 indicate a strong phylogenetic signal, and high values > 15 indicate effectively no signal).

We initially investigated the relationship of body size with our other variables. Log forewing length was therefore entered into COMPARE as the independent (*X*) variable, with the other variable as the dependent (*Y*) variable. When it became apparent that body size was correlated with our other traits (see results), we subsequently controlled for its potential confounding effects on our analysis in the manner advocated by Freckleton (2009)—that is, by including it as a covariate in our other calculations where we related aspects of pheromone titre, molecular weight, and population abundance to antennal morphology (either presence/absence of elaborate antennae or antennal length). The reported PGLS correlations between these characteristics are therefore partial correlations controlling for body size.

Finally, for the species for which we had complete information on pheromone titre, male abundance, and molecular weight, we examined which combination of factors served as the best approximating model of antennal morphology including the model with body size only as predictor. Comparison of models was performed using Akaike's information criterion (AIC_c_) correcting for small sample size (Burnham and Anderson 2002). From the AIC_c_ values, we calculated Akaike weight (*W*_i_) for each model in the candidate set as well as the evidence ratio (ER). The latter provides a means of expressing the relative likelihood of one model over another.

## Results

### Relationship with body size

**Figure 3:**
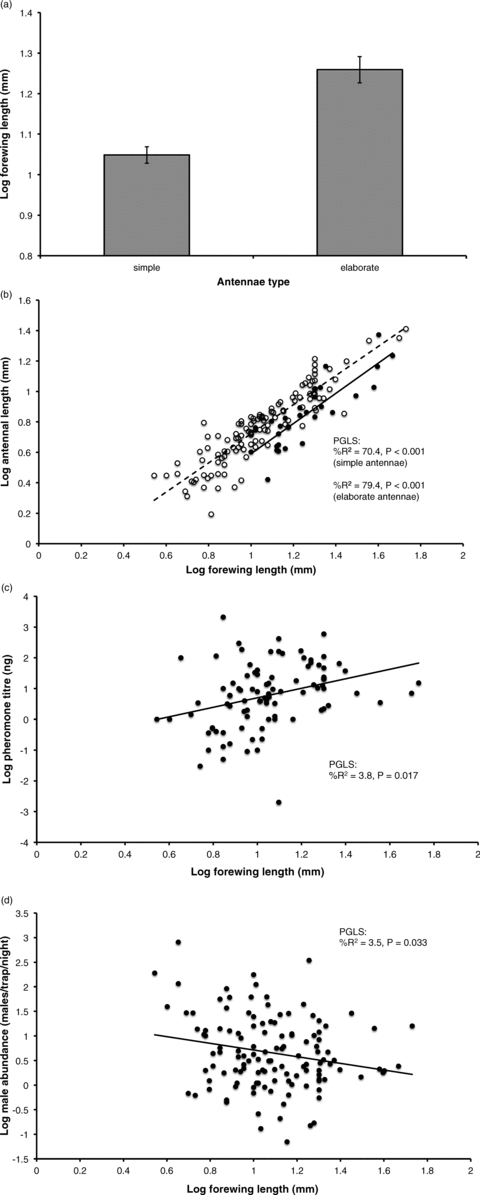
Relationships with body size (log forewing length) and (a) antennal shape (means and standard errors of forewing length shown); (b) log antennal length (open circles, dotted line: simple antennae, filled circles, solid line: elaborate antennae); (c) pheromone titre (log ng pheromone per female); and (d) male abundance (log number of males caught per trap per day).

Body size (measured by forewing length) is significantly linked with antennal morphology. On average, species with elaborate antennae are larger than those with simple antennae (PGLS: α = 9.48, *t*_151_ = 3.339, *P* = 0.001: Fig. 3a). Elaborate antennae are typically shorter, relative to forewing length, than simple antennae (Fig. 3b; average length = 39.9% of forewing length for elaborate antennae, 53.9% of forewing length for simple antennae, *t*_151_ = 5.936, *P* < 0.001). With both types of antennae there is a strong correlation between antennal length and forewing length (Fig. 3b; simple antennae: α = 2.79, *r* = 0.839, *n* = 117, *P* < 0.001, elaborate antennae: α = 3.94, *r* = 0.891, *n* = 30, *P* < 0.001).

Larger bodied species have significantly larger pheromone titres (α = 11.44, *r* = 0.194, *n* = 150, *P* = 0.017: Fig. 3c). However, there was no evolutionary association between mean molecular weight of pheromone components and body size (α = 3.63, *r* = 0.049, *n* = 152, *P* = 0.549). Male abundance (maximum number of individual males caught per trap per night) significantly declined with body size (α = 14.51, *r* = -0.188, *n* = 127, *P* = 0.033: Fig. 3d).

### Relationship with pheromone titre

We found no significant association between pheromone titre and antennae type (i.e., simple or elaborate) after taking phylogeny and body size into account (α = 6.44, *t*_148_ = –0.175, *P* = 0.863). Nor was there an association between pheromone titre and antenna length (species with simple antennae: α = 6.99, *r* = –0.073, *n* = 117, *P* = 0.434; species with elaborate antennae: α = 2.82, *r* = –0.214, *n* = 30, *P* = 0.256).

### Relationship with molecular weight

**Figure 4:**
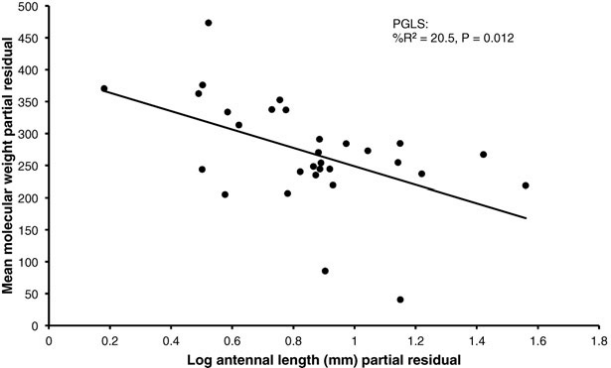
Partial residual plot of the relationship between antennal length and mean molecular weight of female sex pheromone components in moths species with elaborate male antennae.

Initial examination of the data suggested an association between mean molecular weight of compounds and antennae type, although in the opposite direction to that predicted, with species with elaborate antennae using heavier compounds. However, the association was not significant after body size and phylogeny were taken into account (α = 7.21, *t*_150_ = 1.633, *P* = 0.105). There was no association between antennal length and molecular weight for species with simple antennae (α = 6.46, *r* = 0.073, *n* = 119, *P* = 0.430). However, in species with elaborate antennae, there was a significant negative relationship between molecular weight and antennal length (α = 4.46, *r* = –0.453, *n* = 30, *P* = 0.012: Fig. 4).

### Relationship with male population abundance

**Figure 5:**
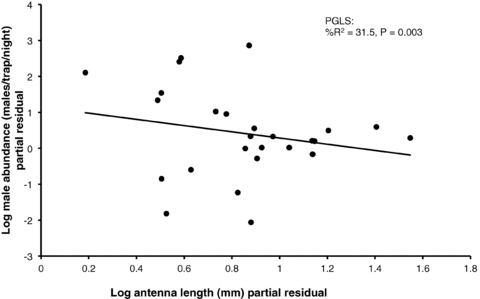
Partial residual plot of the relationship between antennal length and male abundance in moth species with elaborate male antennae.

Although species with elaborate antennae tended to have lower abundance of males, as determined by trapping numbers, there was no significant association between abundance and the type of antenna after controlling for body size and phylogeny (α = 5.63, *t*_125_ = –1.563, *P* = 0.121). However, for species with elaborate antennae, there was a significant negative relationship (Fig. 5) between abundance and antennae length (α = 0.95, *r* = –0.561, *n* = 26, *P* = 0.003). No such pattern was evident in species with simple antennae (α = 8.91, *r* = 0.080, *n* = 97, *P* = 0.436).

### Comparison of models

Evaluation of the AIC_c_ scores for models predicting variation in antennal morphology (simple or elaborate) revealed that the model including both body size and male population abundance was the best approximating model (Table 2a). However, the Akaike weight (0.41) for this model showed that there is only a 41% chance that it was correctly identified as the best approximating model. The ER indicated it was only 1.49 times more likely than the model that included body size alone. The full-factorial model was the least strongly supported model. This was also the case in the comparison of models predicting antennal length in species with simple antennae (Table 2b). In that case, the best approximating model contained only body size, and none of our putative predictors, although the Akaike weights indicated considerable uncertainty in the identity of the best approximating model.

**Table 2:** Comparison of models predicting antennal morphology, and antennal length in species with simple and elaborate antennae, respectively. Models are compared using Akaike's Information Criterion corrected for small sample size (AIC_c_). For other abbreviations see Methods

Model	%*R*^2^	AIC_c_	ΔAIC_c_	*W_i_*	ER
(a) Antennal morphology (*n* = 126)					
Pheromone titre + male abundance + molecular weight + body size	7.79	–137.44	3.57	0.07	5.96
Pheromone titre + body size	3.82	–138.11	2.90	0.10	4.26
Male abundance + body size	5.95	–141.01	0	0.41	1
Molecular weight + body size	5.84	–139.04	1.97	0.15	2.67
Body size only	3.78	–140.21	0.79	0.27	1.49
(b) Antennal length in species with simple antennae (*n* = 97)					
Pheromone titre + male abundance + molecular weight + body size	75.57	–342.98	3.21	0.07	4.98
Pheromone titre + body size	73.93	–345.64	0.55	0.26	1.32
Male abundance + body size	73.98	–344.66	1.53	0.16	2.15
Molecular weight + body size	74.38	–344.92	1.27	0.18	1.84
Body size only	73.44	–346.19	0	0.34	1
(c) Antennal length in species with elaborate antennae (*n* = 26)					
Pheromone titre + male abundance + molecular weight + body size	90.75	–100.50	1.96	0.23	2.66
Male abundance + molecular weight + body size	90.22	–102.46	0	0.62	1
Pheromone titre + body size	82.04	–90.83	11.63	0.00	334.65
Male abundance + body size	87.86	–99.27	3.19	0.13	4.92
Molecular weight + body size	84.80	–95.31	7.15	0.02	35.63
Body size only	81.75	–93.06	9.40	0.01	109.78

By contrast, comparison of models predicting variation in antennal length in species with elaborate antennae (Table 2c) revealed that the model including both male population abundance and molecular weight was estimated to be the best approximating model, with a fairly robust Akaike weight (0.62), although the full-factorial model and the model including only population abundance both stand as credible alternatives. By summing Akaike weights across models, we can estimate predictor weights, which indicate a 98% likelihood that male abundance features in the best approximating model, and an 85% likelihood that molecular weight does as well. Our estimated best approximating model is almost 110 times more likely to be the best model than the model including only body size.

The *R*^2^ values of our full-factorial models predicting antennal shape are extremely poor (7.79%), when compared with the models predicting antennal length (75.57% for species with simple antennae, 90.75% for species with elaborate antennae).

## Discussion

Elaborate antennae in male moths appear have to have evolved at least 13 times, as judged from our phylogeny and sample of species (Fig. 2). Major families in which the characteristic has appeared include the Lymantriidae, Saturniidae, Bombycidae, Geometridae, Cossidae, Sesiidae, and Limacodidae.

The presence of elaborate antennae in moths is closely associated with larger body size (longer forewing length). While antennal length predictably scales with body size in insects (e.g. Emlen and Allen 2004; Bonduriansky 2007), the fact that the gross shape of the antennae also appears to be linked to body size suggests a cost of bearing these antennae that is difficult to sustain for small-bodied species. Elaborate antennae tend to be shorter, relative to forewing length, than simple antennae, suggesting a constraint on antennal length imposed by shape. For example, one cost of elaborate antennae might be related to flight ability. On the one hand, in the large-bodied *Manduca sexta* hawkmoth, elaborate antennae act as mechanosensory gyroscopes, helping to stabilize the animal while hovering (Sane et al. 2007). However, at small body sizes, inertial drag forces associated with having elaborate antennae would be disproportionately higher (Ellington 1991).

These results are also complicated by the use of forewing length as a measure of body size. Typically, moths with longer wings tend to be better at flying over longer distances and for longer periods of time (e.g., Shirai 1993) and hence potentially face lower selection pressure for greater sensitivity to pheromones, because they can compensate for lower sensitivity by airborne searching for longer. In such case, we would be less likely to see a relationship between long forewings and elaborate antennae.

Body size is also linked to pheromone titre: larger species tend to produce greater quantities of pheromone. This relationship between pheromone titre and body size has been identified within several species of moths and may reflect female quality (Jaffe et al. 2007, Johansson and Jones 2007; Harari et al. 2011): females that produce greater quantities of pheromone tend to attract more males (Nagata et al. 1972; Turgeon et al. 1983; Evenden and Gries 2008). Body size is also negatively correlated with male abundance, consistent with other studies of many animal assemblages, including insects (Blackburn et al. 1993; White et al. 2007). The associations of body size with antennal shape and pheromone titre revealed in our analysis indirectly contradict our predictions. If females of small-bodied species produce absolutely smaller amounts of pheromone, then males of these smaller bodied species should be more likely to have elaborate antennae, yet we found the opposite pattern.

However, we found no clear associations between basic antennal shape, or antennal length and pheromone titre after controlling for body size. Female moths typically produce minute quantities of pheromone (Svensson 1996), and males typically possess a greater number of sensilla on their antennae than females (Chapman 1982), suggesting there is a selective advantage for greater sensitivity. However, we cannot find evidence that reduced pheromone titre has selected for increases in antennal size and elaboration. It is important to stress that pheromone titre should ideally be measured as the number of molecules released per unit time (i.e., the release rate) (Hölldobler and Wilson 1990, p. 244), rather than absolute amount in the gland, as we have used here. This was not feasible for the present study because comparative data on release rates for moth species are very sparse. Both pheromone titre and release rates vary within species and within individuals, relating to age, time of day, and proximity of other individuals (e.g., Sanders and Lucuik 1972; Raina et al. 1986; Foster et al. 1995; Lim et al. 2007). Although there is evidence that release rates are limited by pheromone gland titres (Schal et al. 1987), and that pheromone titre is reflective of pheromone release rate (see Methods), it is possible that using fixed quantities of pheromone as a substitute for the quantity released is simply too inaccurate. Additionally, although gas chromatography-mass spectrometry analytically techniques have become increasingly more powerful at detecting minute quantities of chemical components, it is possible that many of the species in our analysis represent the high end of pheromone production rates. Therefore, while we cannot find support for an association, we are reluctant to rule out a relationship between pheromone production and antennal morphology.

Antennal shape was not significantly associated with either the abundance of males or the diffusion rate of the female sex pheromone (as measured by the mean molecular weight of compounds). Nevertheless, the trend is in the direction predicted (more elaborate antennae in species with lower male abundance), and model selection with AIC_c_ suggested that the best model predicting antennal shape included both body size and male abundance. This model was only slightly better supported than the model that included body size alone, and adding male abundance to the model explained only an additional 2% of the variation. Evidently, any effect of male abundance on the evolution of elaborate antennae is difficult to disentangle from the effects of body size.

Nevertheless, variation in antennal length is significantly explained by male abundance and the molecular weight of the female sex pheromone. In species that possess elaborate antennae, there were clear relationships: longer antennae are found in species that have lower male abundances and where the females use pheromone compounds that have, on average, lower molecular weights (i.e., that diffuse and fade out more quickly). Model selection with AIC_c_ demonstrated much stronger support for models that included these predictors than the model that included body size alone (see Table 2c). This result is interesting because antennal length is only one component of antennal size and there is considerable variation in elaborate antennae area apart from length. Some species (e.g., *Agrotis ipsilon*) have only slightly ramified antennae compared to the broad width on others such as *Coloradia velda*. Additionally, most of the species in our analysis are agricultural pests whose population sizes are likely to have increased enormously since the introduction of intensive agricultural practices in the past few hundred years. Present measures of abundance through trapping data may therefore be unreflective of the population dynamics under which the species (and their antennae) evolved. Having said this, changes in abundance should apply to all species (i.e., they have all increased in abundance recently), so we doubt any systematic bias in our analysis. More pertinently, despite all these potential inaccuracies and the possible confounding effects of forewing length and flight ability (see earlier), the strong pattern (model *R*^2^ > 90%) observed here relating antennal length to abundance and molecular weight strikes us as being biologically significant.

It is noteworthy that these patterns apply to species with elaborate antennae only and not to species with simple antennae. Given the approximately one-dimensional nature of a filiform antenna, an increase in length of antenna would have less effect on the number of sensilla than it would on a more two- or three-dimensional feathery antenna. Specifically, in the latter cases, an increase in the length of antenna would result in either a squaring or cubing of the antennal surface area, rather than the simple isometric increase that would occur in species with filiform antennae. In species with filiform antennae, greater sensitivity might be achieved by developing longer sensilla (rather than longer antennae per se), as noted in the cabbage looper moth, *Trichoplusia ni* (O’Connell et al. 1983).

Our analyses necessarily trade-off collating data from a sufficient sample of species, with data quality–with considerable noise deriving from factors that influence the dispersion of pheromones (plume structure and environmental turbidities) and population abundance (see earlier). Additionally, as our understanding of moth phylogenetic relationships becomes clearer, the interpretation of how often, and why, elaborate antennae have evolved is likely to be modified. Nevertheless, we can derive two main conclusions about the evolution of antennae in moths. First, the elaborate male antennae borne by some moth species are associated with larger body size, suggesting a cost to these structures. Accordingly, there must be equally strong benefits, that we would hypothesize most likely derive from sexual selection for this type of antennal morphology. Second, small pheromone titres, low male abundance, and diffusion properties of the pheromone do not directly account for why males of some species have simple antennae and others elaborate antennae. However, the latter two factors may influence the size of those structures in species that have elaborate antennae. More generally, it is evident that other factors, perhaps associated with reproductive ecology and mating system, may select for the evolution of these remarkable lepidopteran characteristics.
